# Influence of the COVID‐19 Pandemic on the Prevalence of Depression Among US Adults

**DOI:** 10.1155/carj/2846550

**Published:** 2026-04-12

**Authors:** Jixiang Liu, Yichong Wang, Ning Ding, Shengsheng Zhang

**Affiliations:** ^1^ Graduate School, Beijing University of Chinese Medicine, No. 11 North 3rd Ring East Road Chaoyang, Beijing, 100029, China, bucm.edu.cn; ^2^ Department of Gastroenterology, Beijing Traditional Chinese Medicine Hospital, Capital Medical University, Dongcheng District, Beijing, China, ccmu.edu.cn

**Keywords:** COVID-19, depression, pandemic

## Abstract

**Background:**

The prevalence of depressive symptoms increased significantly in the early stages of the COVID‐19 pandemic and remained elevated even as lockdown policies were eased. Therefore, a thorough evaluation of the pandemic’s long‐term effects on depressive disorders is critical.

**Methods:**

A repeated cross‐sectional study was conducted using data from the National Health Interview Survey collected before (2017–2018) and during the later stage of the COVID‐19 pandemic (August 2021−August 2023). *χ* ^2^ tests were conducted to compare the characteristics between nondepressed and depressed adults. Weighted multivariable logistic regression models were employed to estimate the associations between social and physical factors with depressive symptoms.

**Results:**

The prevalence of depressive symptoms in the later stages of the COVID‐19 pandemic (12.7%) was significantly higher than prepandemic levels (9.1%). Specifically, the incidence rates for mild, moderate, moderately severe, and severe depressive symptoms increased by 1.24‐fold, 1.44‐fold, 1.38‐fold, and 1.71‐fold, respectively, compared to prepandemic levels. Higher risk of developing depressive disorders persisted among those who were widowed/divorced/separated, never married, and with lower education or income. Prepandemic protective factors such as lower systemic inflammatory response index, no smoking or drinking, and no hypertension history became less effective. Males and females aged 20–40 exhibited the highest probability of developing depressive symptoms in the later stage of the pandemic.

**Limitations:**

The cross‐sectional design prevents inference of causality.

**Conclusions:**

The persistence of mental health disparities highlights the need for sustained efforts from the government, mental healthcare organizations, and healthcare providers to support vulnerable groups even beyond the pandemic.

## 1. Introduction

Depression is a common mental disorder characterized by persistent sadness, a loss of interest or pleasure, and a variety of emotional and physical symptoms. It has been found that depression is closely linked to alexithymia and suicidal ideation, significantly impacting individuals’ life quality and overall well‐being [1, 2]. The acute emergence of the coronavirus disease 2019 (COVID‐19) pandemic, along with the subsequent implementation of lockdown policies, financial market instability, and concerns about physical health, heightened population vulnerability, particularly among individuals with preexisting cognitive dysfunctions, to stressful circumstances [3]. This contributed to a nearly twofold increase in the prevalence of self‐reported depression among US adults, reaching 25.8% between March and April 2020 [4]. Notably, previous studies found that, unlike other mental disorders, the prevalence of depression did not return to prepandemic levels even as lockdown measures were eased. Instead, it showed a small but statistically significant increase [5]. Therefore, the depression conditions during the later stage of the pandemic have been highlighted as a cause for concern.

Several studies have examined the trajectory of self‐reported depressive symptoms during the early stages of the pandemic. For instance, a meta‐analysis that aggregated data from sixty‐five longitudinal cohort studies found that depressive symptoms significantly worsened during the early months (March−April 2020) and showed a persistently slight increase in the subsequent months (May−July 2020) [5]. A similar trend of a sustained but slight rise in depression prevalence was also observed among UK adults from July 2020 to March 2021 [6]. Both studies emphasized the severity of depressive symptoms during the pandemic’s early phases and stressed the importance of ongoing monitoring. As vaccinations progressed during the summer of 2021, COVID‐19 cases gradually declined [7]. However, there have been fewer studies focusing on the long‐term effects of the pandemic on depressive symptoms since then. Only a recent study conducted in Switzerland with a two‐year follow‐up found that the severity of depressive symptoms in the later stage of the pandemic had significantly decreased compared to those observed in the early stage of the pandemic [8]. Nevertheless, due to the lack of prepandemic baseline data, no studies have definitively determined whether depressive symptoms in the later stage of the pandemic have returned to prepandemic levels. Thus, a comprehensive evaluation of the long‐term impacts of the pandemic on depressive disorders remains necessary.

The COVID‐19 pandemic has been reported to have disproportionate impacts on different age and sociodemographic groups via its direct psychological effects and long‐term economic and social consequences [9, 10]. For instance, compared to men and older adults, women and younger adults were more likely to have depressive symptoms during the early stages of the pandemic [11, 12], potentially due to their higher stress vulnerability or more significant disruptions in daily life. In addition, Black, Hispanic, and Asian respondents have experienced relatively worse outcomes compared to White respondents [13]. However, there is limited research focusing on the subgroup with existing physical health conditions during the pandemic, such as obesity and chronic diseases. Thus, further identifying and monitoring the susceptible individuals are essential.

This study aims to assess the impact of the COVID‐19 pandemic on depressive symptoms among US adults by comparing Patient Health Questionnaire‐9 (PHQ‐9) data from the National Health and Nutrition Examination Survey (NHANES) across the pre‐COVID‐19 pandemic (2017–2018) and post‐COVID‐19 pandemic (2021–2023) periods. Furthermore, by examining how the sociodemographic and health‐related characteristics of individuals with depression have shifted across these time points, the study seeks to identify population subgroups most vulnerable to depression under the acute stress of the pandemic. We hypothesize that (1) the prevalence and severity of depressive symptoms will be significantly higher during the later pandemic period compared to the prepandemic period, and (2) sociodemographic and health‐related risk profiles associated with depression will shift during the pandemic, with heightened vulnerability observed among individuals with lower socioeconomic status, disrupted social support (e.g., unmarried or socially isolated individuals), and preexisting health disparities.

## 2. Methods

This repeated cross‐sectional study utilized a nationally representative sample derived from the NHANES conducted by the US National Center for Health Statistics. NHANES has been systematically collecting biennial data since 1999 to provide a detailed profile of the health and nutritional status of the US civilian noninstitutionalized population. The survey employs a complex, multistage probability sampling design, incorporating state, county, census tract, and household levels to ensure the representation of diverse demographic groups. Key features of this design include oversampling of specific subpopulations, adjustments for nonresponse, and stratification, which collectively necessitate the application of sample weights in statistical analyses to produce unbiased estimates. Each participant was recruited for an in‐depth, in‐person interview and a comprehensive set of physical examinations and laboratory assessments conducted in a mobile examination center. The NHANES study protocol has been reviewed and approved by the National Center for Health Statistics Institutional Review Board, and all participants provided written informed consent before participating.

### 2.1. Study Population

We restricted our analysis to adults aged 20 years and older who participated in NHANES cycles during the pre‐COVID‐19 pandemic (2017‐2018) and post‐COVID‐19 pandemic (August 2021–August 2023) periods. Among these participants (*n* = 13,378), any individual who was pregnant (*n* = 96) or missing data on the depression questionnaire (*n* = 3328) was excluded from the analysis. Finally, a total of 9554 adults were enrolled in the final analysis. In this study, we define the “pre‐COVID‐19 period” as the time before the COVID‐19 pandemic and the “post‐COVID‐19 period” as the later stage of the COVID‐19 pandemic.

### 2.2. Assessment of Depressive Symptoms

Depressive symptoms in adults were assessed using the PHQ‐9, a widely validated self‐report tool. The instrument consists of nine items, each rated from 0 to 3, yielding a total score between 0 and 27. Based on established cutoffs, scores were categorized as follows: no (0–4), mild (5–9), moderate (10–14), moderately severe (15–19), and severe (≥ 20) depression.

### 2.3. Covariates

Covariates included sociodemographic characteristics, anthropometric and hematological measurements, lifestyle behaviors, and long‐term health conditions. Sociodemographic characteristics, such as gender (male or female), age (20–39, 40–59, and ≥ 60), race and ethnicity (non‐Hispanic White, non‐Hispanic Black, non‐Hispanic Asian, Mexican American, and other), educational attainment (less than high school, high school or general equivalency diploma, and college or above), marital status (married/living with partner, widowed/divorced/separated, and never married), and family poverty income ratio (≤ 1.3, 1.3–3.5, and > 3.5), were collected using standardized questionnaires.

Anthropometric measurements included body mass index (BMI), which was used to assess obesity levels and was categorized into three groups: underweight or normal weight (< 25.0), overweight (25.0–29.9), and obese (≥ 30). Hematological measurements included the systemic inflammatory response index (SIRI), which was employed to evaluate the inflammation level in adults. SIRI was calculated using the formula: SIRI = (neutrophil count × monocyte count)/lymphocyte count. The counts of neutrophils, monocytes, and lymphocytes were obtained through blood analysis using a quantitative automated hematology analyzer (UniCel DxH 800). The SIRI was categorized according to the tertiles: low (< 0.80), intermediate (≥ 0.80 and < 1.32), and high (≥ 1.32).

Self‐reported lifestyle factors included smoking status (never and history of smoking) and alcohol use (never and history of drinking). Long‐term health conditions refer to the medical history of the subjects. Diabetes was ascertained based on self‐reported diagnosis, current use of insulin or antidiabetic medications, or meeting any of the following criteria: fasting plasma glucose levels ≥ 7.0 mmol/L, 2 h postprandial plasma glucose levels ≥ 11.1 mmol/L, or glycated hemoglobin A1c (HbA1c) ≥ 6.5%. Hypertension was defined by self‐reported diagnosis, ongoing use of antihypertensive medications, or meeting any of the following criteria: systolic blood pressure ≥ 140 mmHg or diastolic blood pressure ≥ 90 mmHg.

### 2.4. Statistical Analysis

According to NHANES analytic guidelines, all analyses in this study incorporated sample weights, clustering, and stratification to estimate appropriate variance and ensure national representation of US adults. Adults were divided into two groups according to the PHQ‐9 score: 0–9 (*nondepression*) and ≥ 10 (*depression*). Baseline characteristics were described across different levels of depressive symptoms. Data were presented as amounts and percentages for categorical variables. *χ* ^2^ tests were conducted to compare characteristics between nondepressed and depressed adults, as well as to assess changes in these characteristics among depressed adults across the pre‐COVID‐19 and post‐COVID‐19 periods. In addition, changes in the prevalence of varying levels of depressive symptoms were calculated for US adults between these two periods. Multivariable logistic regression models were applied to estimate odds ratios (ORs) and 95% CI for the associations between social and physical factors with depressive symptoms; the model was controlled for gender, age, and race/ethnicity. To assess the nonlinear relationship between age, gender, and the prevalence of depression during different COVID‐19 occurrence phases, smoothed splines were used.

## 3. Results

### 3.1. Baseline Characteristics

A total of 9954 adults were included in the analysis: 4779 adults (age = 48.5 ± 0.6 years [mean ± SE]; 49.0% males) from the pre‐COVID‐19 period and 5175 adults (age = 49.0 ± 0.6 years [mean ± SE]; 49.6% males) from the post‐COVID‐19 period. Participants′ characteristics are presented in Table 1 according to PHQ‐9 scores. 435 participants (9.1%) had depressive symptoms in the pre‐COVID‐19 period and 657 participants (12.7%) in the post‐COVID‐19 period. In general, the prevalence of depressive symptoms increased by approximately 1.4‐fold in the post‐COVID‐19 period compared to the pre‐COVID‐19 period.

**TABLE 1:** Demographic characteristics of US adults in the pre‐COVID‐19 and post‐COVID‐19 periods.

Characteristic	Pre‐COVID‐19 period^a^ No. (%)		*p* value^d^	Post‐COVID‐19 period^b^ No. (%)		*p* value^d^	*p* value^e^
	Total	Depression^c^		Total	Depression^c^		
Overall	4779	435 (9.1)		5175	657 (12.7)		
Age (years)			0.660			< 0.001	0.061
20–39	1418 (35.1)	125 (8.8)		1303 (34.8)	240 (18.4)		
40–59	1539 (35.6)	144 (9.4)		1478 (33.5)	194 (13.1)		
≥ 60	1822 (29.3)	166 (9.1)		2394 (31.7)	223 (9.3)		
Gender			0.006			< 0.001	0.894
Male	2359 (49.0)	175 (7.4)		2364 (49.6)	243 (10.3)		
Female	2420 (51.0)	260 (10.7)		2811 (50.4)	414 (14.7)		
Race and ethnicity			0.008			0.008	0.996
Mexican American	639 (8.7)	163 (25.5)		328 (6.7)	46 (14.0)		
Non‐Hispanic White	1693 (63.2)	490 (28.9)		3197 (62.7)	383 (12.0)		
Non‐Hispanic Black	1123 (11.2)	271 (24.1)		590 (10.5)	83 (14.1)		
Non‐Hispanic Asian	640 (5.4)	89 (13.9)		228 (5.1)	17 (7.5)		
Other	248 (11.5)	85 (34.3)		339 (15.0)	128 (37.8)		
Body mass index (kg/m^2^)			0.005			0.031	0.236
< 25	1206 (26.1)	88 (7.3)		1355 (27.1)	152 (11.2)		
25−30	1514 (30.8)	106 (7.0)		1651 (32.3)	180 (10.9)		
≥ 30	2009 (43.0)	232 (11.5)		2123 (40.6)	310 (14.6)		
Marital status			< 0.001			< 0.001	0.004
Married/living with partner	2817 (62.6)	186 (6.6)		2795 (60.0)	249 (8.9)		
Widowed/divorced/separated	1094 (18.7)	157 (14.4)		1296 (18.7)	195 (15.0)		
Never married	863 (18.8)	91 (10.5)		1080 (21.3)	212 (19.6)		
Educational attainment			< 0.001			0.001	0.327
Some college or above	2723 (61.9)	206 (7.6)		3567 (65.9)	398 (11.2)		
High school graduate or general equivalency diploma	1674 (34.6)	178 (10.6)		1430 (31.4)	223 (15.6)		
Less than high school graduate	376 (3.4)	49 (13.0)		176 (2.7)	35 (19.9)		
Poverty income ratio			< 0.001			< 0.001	0.507
> 3.5	1291 (46.0)	61 (4.7)		1942 (46.3)	136 (7.0)		
1.3−3.5	1509 (33.7)	125 (8.3)		1503 (34.4)	211 (14.0)		
≤ 1.3	1166 (20.3)	156 (13.4)		936 (19.3)	208 (22.2)		
SIRI			0.075			0.928	0.067
Low	1542 (30.2)	126 (8.2)		1608 (34.0)	201 (12.5)		
Medium	1525 (34.7)	125 (8.2)		1680 (33.3)	205 (12.2)		
High	1532 (35.2)	161 (10.5)		1649 (32.7)	217 (13.2)		
Smoking status			< 0.001			0.008	0.001
Never smoke	2734 (57.2)	185 (6.8)		3025 (61.8)	329 (10.9)		
History of smoke	2045 (42.8)	250 (12.2)		2143 (38.2)	327 (15.3)		
Alcohol drinking status			0.002			0.878	0.002
Never drink	483 (7.1)	25 (5.2)		441 (8.5)	61 (13.8)		
History of drink	4293 (92.9)	410 (9.6)		4710 (91.5)	593 (12.6)		
History of diabetes			0.602			0.926	0.145
Yes	2526 (62.6)	244 (9.7)		2459 (59.1)	300 (12.2)		
No	983 (37.4)	92 (9.4)		1357 (40.9)	163 (12.0)		
History of hypertension			< 0.001			0.346	< 0.001
Yes	1820 (32.9)	217 (11.9)		1945 (31.2)	260 (13.4)		
No	2950 (67.1)	218 (7.4)		3228 (68.8)	397 (12.3)		

Abbreviations: COVID‐19, coronavirus disease 2019; SIRI, systemic inflammatory response index.

^a^Pre‐COVID‐19 period, estimates were derived from the National Health and Nutrition Examination Survey (NHANES) for the years 2017–2018.

^b^Post‐COVID‐19 period, estimates were derived from the National Health and Nutrition Examination Survey (NHANES) for the years 2021–2023.

^c^Defined as Patient Health Questionnaire‐9 score of 10 or greater.

^d^Two‐tailed *χ* ^2^ analysis was used to compare nondepression and depression in adults.

^e^Two‐tailed *χ* ^2^ analysis was used to compare depression in adults before and during the later stage of the COVID‐19 pandemic.

The distribution of depressive symptoms across several demographic categories in the post‐COVID‐19 period remained consistent with pre‐COVID‐19 pandemic patterns. For instance, participants who were female, obese, widowed/divorced/separated, never married, had lower educational attainment, lower income levels, and a history of smoking were more likely to experience depression. These associations were evident both in the prepandemic and postpandemic periods. However, there are still some differences in the depression prevalence among subgroups of individuals between the pre‐COVID‐19 and post‐COVID‐19 periods. Specifically, there was no significant variation in the prevalence of depression across different age groups in the pre‐COVID‐19 period. However, individuals aged 20–40 have shown a notably higher prevalence of depression compared to other age groups during the postpandemic period. Among participants with depression, significant differences in demographic characteristics, such as marital status, smoking habits, alcohol consumption, and history of hypertension, were observed between the pre‐COVID‐19 and post‐COVID‐19 periods.

### 3.2. Comparison of the Prevalence of Depressive Symptoms in the Pre‐COVID‐19 and Post‐COVID‐19 Periods

The prevalence of depressive symptoms during the post‐COVID‐19 period was significantly higher across all severity levels compared to the pre‐COVID‐19 period (Table 2). Specifically, the prevalence of mild, moderate, moderately severe, and severe depressive symptoms increased by 3.8%, 2.5%, 0.8%, and 0.5%, representing 1.24, 1.44, 1.38, and 1.71 times higher than prepandemic levels.

**TABLE 2:** Prevalence of depressive symptoms among US

Depressive symptoms^a^	No. (weighted %)		Difference		*p* value^d^
	Pre‐COVID‐19 period^b^	Post‐COVID‐19 period^c^	Absolute, %	Relative	
None	3556 (75.3)	3491 (67.9)	−7.4	0.90	< 0.001
Mild	788 (16.1)	1027 (19.9)	3.8	1.24	
Moderate	279 (5.7)	419 (8.2)	2.5	1.44	
Moderately severe	114 (2.1)	170 (2.9)	0.8	1.38	
Severe	42 (0.7)	68 (1.2)	0.5	1.71	

*Note:* Adults in the pre‐COVID‐19 and post‐COVID‐19 periods.

Abbreviation: COVID‐19, coronavirus disease 2019.

^a^Depressive symptoms assessed using the Patient Health Questionnaire‐9 and categorized as none (score: 0–4), mild (score: 5–9), moderate (score: 10–14), moderately severe (score: 15–19), and severe (score: ≥ 20).

^b^Pre‐COVID‐19 period, estimates were derived from the National Health and Nutrition Examination Survey (NHANES) for the years 2017–2018.

^c^Post‐COVID‐19 period, estimates were derived from the National Health and Nutrition Examination Survey (NHANES) for the years 2021–2023.

^d^Two‐tailed *χ* ^2^ analysis was used to conduct significance testing.

### 3.3. Association Between Covariates and Depressive Symptoms in the Pre‐COVID‐19 and Post‐COVID‐19 Periods

After adjusting for covariates, individuals who were widowed/divorced/separated, never married, had lower educational attainment, and lower income remained at higher risk for depression both in the pre‐COVID‐19 and post‐COVID‐19 periods (Table 3). Notably, never‐married participants exhibited a higher risk of depressive symptoms in the post‐COVID‐19 period (OR: 1.67; 95% CI: 1.08–2.60) compared to the pre‐COVID‐19 period (OR: 1.98; 95% CI: 1.48–2.66). Before the pandemic, individuals with a lower SIRI, who had never smoked, never consumed alcohol, or had no history of hypertension, were associated with a reduced risk of developing depression. However, these protective associations markedly diminished or vanished during the post‐COVID‐19 period.

**TABLE 3:** Factors associated with depressive symptoms among US

Factors	Pre‐COVID‐19 period^a^		Post‐COVID‐19 period^b^	
	OR (95% CI)^c^	*p* value	OR (95% CI)^c^	*p* value
Body mass index (kg/m^2^)				
< 25	1 [reference]		1 [reference]	
25−30	0.67 (0.43, 1.05)	0.060	0.99 (0.75, 1.30)	0.930
≥ 30	1.11 (0.72, 1.71)	0.552	1.33 (0.94, 1.86)	0.164
Marital status				
Married/living with partner	1 [reference]		1 [reference]	
Widowed/divorced/separated	2.40 (1.59, 3.62)	0.002	2.03 (1.59, 2.61)	0.001
Never married	1.67 (1.08, 2.60)	0.023	1.98 (1.48, 2.66)	0.004
Educational attainment				
Some college or above	1 [reference]		1 [reference]	
High school graduate or general equivalency diploma	1.44 (1.06, 1.96)	0.021	1.50 (1.22, 1.86)	0.013
Less than high school graduate	2.44 (1.36, 4.35)	0.008	2.27 (1.39, 3.70)	0.022
Poverty income ratio				
> 3.5	1 [reference]		1 [reference]	
1.3−3.5	1.76 (1.26, 2.46)	0.021	2.00 (1.52, 2.63)	0.004
≤ 1.3	3.76 (2.66, 5.31)	< 0.001	3.44 (2.60, 4.55)	< 0.001
SIRI				
High	1 [reference]		1 [reference]	
Medium	0.69 (0.51, 0.92)	0.013	1.01 (0.75, 1.35)	0.949
Low	0.73 (0.55, 0.98)	0.037	0.97 (0.71, 1.31)	0.838
Smoking status				
History of smoke	1 [reference]		1 [reference]	
Never smoke	0.31 (0.22, 0.44)	< 0.001	0.65 (0.43, 1.00)	0.064
Alcohol drinking status				
History of drink	1 [reference]		1 [reference]	
Never drink	0.45 (0.26, 0.79)	0.012	1.09 (0.70, 1.69)	0.716
History of diabetes				
Yes	1 [reference]		1 [reference]	
No	0.81 (0.65, 1.01)	0.077	0.89 (0.71, 1.11)	0.302
History of hypertension				
Yes	1 [reference]		1 [reference]	
No	0.44 (0.34, 0.55)	< 0.001	0.70 (0.57, 0.87)	0.004

*Note:* Adults in the pre‐COVID‐19 and post‐COVID‐19 periods.

Abbreviations: COVID‐19, coronavirus disease 2019; SIRI, systemic inflammatory response index.

^a^Pre‐COVID‐19 period, estimates were derived from the National Health and Nutrition Examination Survey (NHANES) for the years 2017–2018.

^b^Post‐COVID‐19 period, estimates were derived from the National Health and Nutrition Examination Survey (NHANES) for the years 2021–2023.

^c^Model controls for demographic characteristics (i.e., gender, age, and race/ethnicity).

### 3.4. Association Between Age and Depression Stratified by Sex in the Pre‐COVID‐19 and Post‐COVID‐19 Periods

Before the pandemic, the prevalence of depressive disorders in both males and females did not vary by age, as shown in Figure 1. However, during the post‐COVID‐19 period, an inverse relationship between age and the probability of developing depressive disorders was observed in males aged 20–43 or 52–71, as well as in females. Notably, among males aged 43–52 and 71–80, the probability of developing depressive disorders increased slightly with age.

FIGURE 1Prevalence of depressive symptoms in the pre‐COVID‐19 and post‐COVID‐19 periods, stratified by age and sex. (a) Males. (b) Females.

**Figure:**
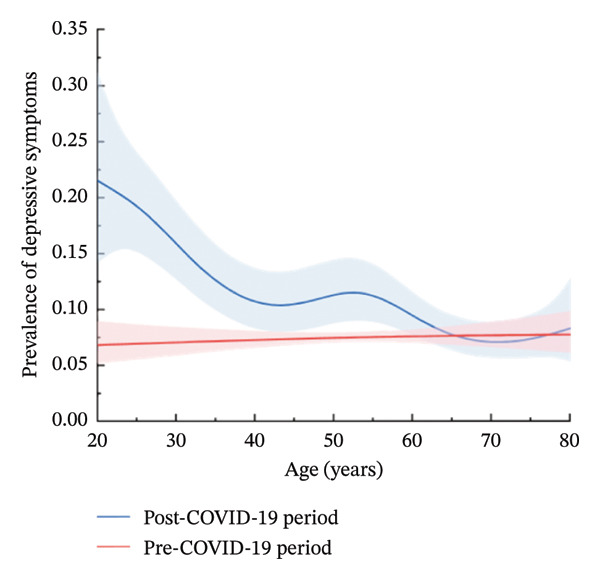
(a)

**Figure:**
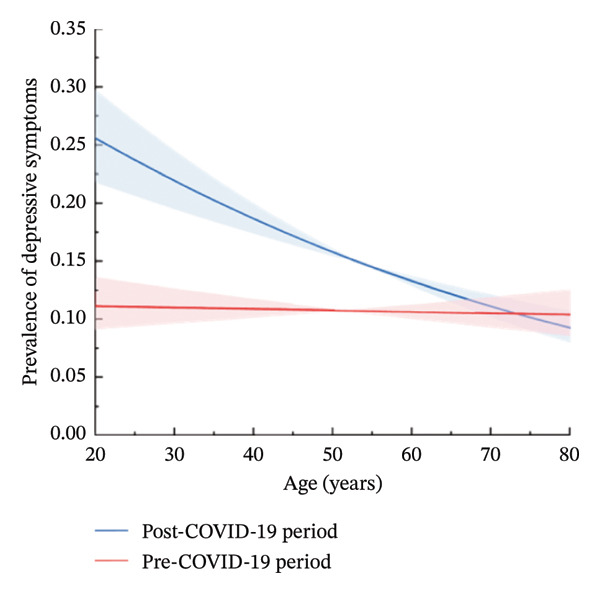
(b)

Furthermore, in comparison to the pre‐COVID‐19 period, the probability of developing depressive disorders in the post‐COVID‐19 period was higher for males under 65 years old and females under 73 years old, with the most significant increases observed among both males and females aged 20–40.

## 4. Discussion

This study revealed a significant increase in depression prevalence among US adults during the later pandemic period, with severe depression rising most markedly to 1.71 times prepandemic levels. After adjusting for gender, age, and race, individuals who were widowed/divorced/separated, never married, less educated, or lower income consistently showed higher depression risk both before and after the pandemic onset. Notably, prepandemic protective factors, including nonsmoking, alcohol abstinence, lower SIRI, and absence of hypertension, were substantially weakened or lost during the pandemic. In addition, depression risk increased significantly among males under 65 and females under 73 after the pandemic, particularly within the 20–40 age group.

Our findings align with prior literature documenting elevated depression rates during and after traumatic events [14]. Specifically, while depression prevalence in the later stages of the COVID‐19 pandemic (12.7%) remained marginally higher than the prepandemic baseline (9.1%), nevertheless, compared to the prevalence of depression among the US population reported in the early stages of the pandemic (27.8%) [8], the rate observed in the later stages (12.7%) shows a significant decline. These observations suggest a general trend toward mental health recovery following the acute phase of the pandemic, highlighting population‐level resilience amid prolonged collective stress.

Our findings corroborate existing evidence that individuals with lower socioeconomic status exhibited an elevated risk of depression both before and after the COVID‐19 pandemic. This heightened vulnerability may stem from multifaceted stressors, including economic precarity, job insecurity, and challenges in reintegrating into daily life amid ongoing uncertainty [15]. Moreover, growing awareness of macrolevel crises, such as pandemic‐related inflation, the conflict in Ukraine, and intensifying climate impacts, may have compounded psychological distress in this population [16–18]. Furthermore, unmarried individuals showed a slightly elevated depression prevalence in the postpandemic period, likely reflecting their increased exposure to loneliness and limited social support during collective trauma [19, 20]. Notably, while better physical health was associated with reduced depression risk before the pandemic, this protective effect substantially weakened or disappeared afterward. This shift suggests that widespread societal disruption during the pandemic may have attenuated the buffering role of individual health advantages. In summary, during the pandemic, the impact of structural social vulnerabilities, such as low income, low education, and nonmarital status, on depression risk remained pronounced, while traditional protective factors such as healthy behaviors and low inflammation levels diminished in effectiveness under systemic societal stress.

Our findings found that the impact of the pandemic on mental health demonstrates distinct patterns across different sociodemographic contexts, particularly by age. In our study, younger adults exhibited a higher probability of developing depression during the later stage of the pandemic compared to those aged 40 and above. This increased vulnerability aligns with the heightened stress sensitivity often observed in younger populations, particularly amid contexts of economic instability [21]. Furthermore, we observed a modest rise in depression probability among males aged 43–52 and 71–80, which may reflect distinct age‐related stressors: middle‐aged men often face employment‐related pressures, while older men may experience elevated anxiety related to mortality risk following infection [22].

This study systematically demonstrates how the prolonged COVID‐19 pandemic structurally altered depression prevalence and risk profiles among US adults. The significant increase in depression prevalence during the later phase of the COVID‐19 pandemic, the attenuation of conventional protective factors, and the persistence of structural social risk factors together indicate that institutional and governmental support may be more consequential than individual health behaviors alone. At the governmental level, the federal government should enhance financial security through measures such as debt relief and eviction protection, while also expanding essential services including childcare and daytime support for older adults. These efforts can help restore confidence in social stability and development among at‐risk populations [10]. At the societal level, mental healthcare organizations should offer accessible and affordable mental health services, along with programs aimed at mitigating loneliness and social isolation [23]. Meanwhile, healthcare providers should place greater emphasis on patients identified via self‐reported screening tools and ensure they receive appropriate follow‐up care if symptoms persist [24]. For the general public, during major societal crises, it is important to acknowledge emotional changes calmly, avoid self‐blame or stigma, better understand how social conditions affect mental health, stay informed about supportive policies and resources, and actively participate in community advocacy. In summary, the findings of this study may provide a reference for other countries and regions experiencing similar public health crises or prolonged societal stress.

### 4.1. Strengths and Limitations

The main strength of this study lies in comprehensively assessing the impact of the pandemic on the prevalence of depression among US adults and identifying characteristics of populations susceptible to depression during the pandemic based on extensive demographic and health information. This study has several limitations that warrant consideration. First, the cross‐sectional design of the data restricts causal inference regarding the observed associations. While the pandemic has been linked to adverse mental health outcomes through both direct psychological impact and longer‐term socioeconomic consequences [25], the temporal and directional nature of these relationships cannot be definitively established from our analysis. Second, although the PHQ‐9 is a widely validated and commonly used screening instrument for depressive symptoms, it reflects self‐reported symptom severity and does not equate to formally diagnosed clinical depression, the prevalence of which is typically lower in population‐based studies. Third, while we examined a range of sociodemographic and health‐related correlates of depression, important psychological constructs such as personality traits, coping styles, and resilience factors, which are known to significantly influence longer‐term mental health adaptation and outcomes [26], were not available in the dataset and thus could not be accounted for in our analyses. Fourth, the study primarily relied on self‐reported questionnaire data, which may be subject to recall bias and social desirability effects, thereby potentially affecting the accuracy of the results. Future longitudinal studies incorporating such psychological measures would be valuable to further elucidate the mechanisms underlying mental health trajectories following major population stressors.

## 5. Conclusions

During the later stages of the pandemic, the prevalence of depression among US adults was significantly higher compared to the prepandemic period. Traditional protective factors against depression, such as healthy behaviors and low inflammation levels, showed reduced effectiveness, while structural social risk factors including low income and limited education remained consistently influential. These findings highlight that during major societal crises, governmental policies and institutional support are critical for protecting the population′s mental health.

## Author Contributions

Jixiang Liu: writing−review and editing, writing−original draft, software, methodology, formal analysis, data curation, and conceptualization. Yichong Wang: writing−review and editing, methodology, and conceptualization. Ning Ding: writing−review and editing, methodology, and conceptualization. Shengsheng Zhang: final approval of the version to be submitted.

## Funding

This research was supported by the National Administration of Traditional Chinese Medicine’s “Hundred Thousand Ten Thousand” Talent Inheritance and Innovation Project (Qi Huang Scholars) National Leading Talent Support Plan for Traditional Chinese Medicine (Grant no. [2021]203 of the Ministry of Traditional Chinese Medicine’s Teacher Education and Personnel Work).

## Conflicts of Interest

The authors declare no conflicts of interest.

## Data Availability

Data are available on the website of the Center for Diseases Control and Prevention (CDC): https://wwwn.cdc.gov/nchs/nhanes/Default.aspx.
